# Minimally Invasive Surgery Versus Medical Management for Spontaneous Supratentorial Intracerebral Hemorrhage: An Updated Systematic Review and Meta-Analysis of Randomized and Propensity Score–Matched Studies

**DOI:** 10.3390/medicina61122216

**Published:** 2025-12-16

**Authors:** Mohammed Maan Al-Salihi, Maryam Sabah Al-Jebur, Ram Saha, Ahmed Saleh, Ahmed Abd Elazim, Farhan Siddiq, Ali Ayyad, Adnan I. Qureshi

**Affiliations:** 1 Zeenat Qureshi Stroke Institute and Department of Neurology, University of Missouri, Columbia, MO 65212, USA; 2 Department of Neurological Surgery, School of Medicine and Public Health, University of Wisconsin, Madison, WI 53705, USA; 3 Department of Neurology, Virginia Commonwealth University, Richmond, VA 23284, USA; 4 Department of Neurosurgery, University of Arizona, Tucson, AZ 85721, USA; 5 Department of Neurology, University of South Dakota, Sioux Falls, SD 57107, USA; 6 Department of Neurosurgery, University of Missouri, Columbia, MO 65212, USA; 7 Department of Neurosurgery, Jordan University Hospital, Amman 11942, Jordan

**Keywords:** minimally invasive surgery, medical therapy, functional outcomes, mortality, meta-analysis

## Abstract

*Background and Objectives:* Minimally invasive surgery (MIS) has emerged as a less invasive alternative to medical management (MM) in intracerebral hemorrhage (ICH), but its comparative effectiveness remains uncertain. *Materials and Methods:* We searched PubMed, Web of Science, Scopus, and Cochrane Library for RCTs and prospective matched studies comparing MIS with MM in supratentorial spontaneous ICH. Primary outcomes were functional recovery and mortality; secondary outcomes were adverse events, rebleeding, and ICU stay. Meta-analysis was performed using RevMan 5.4 with 95% confidence intervals (CI). *Results:* Thirteen studies (11 RCTs, 2 cohorts; 1503 MIS, 1401 MM) were included. MIS significantly improved functional outcomes (Risk Ratio, RR 1.18, 95% CI 1.01 to 1.38), driven mainly by studies including both deep and lobar hematomas, whereas deep-only studies showed inconsistent effects. The benefit was largely attributable to stereotactic aspiration with local thrombolytics (RR 1.23, 95% CI 1.07 to 1.41), while other MIS techniques showed no significant advantage. Early intervention (<24 h) demonstrated better outcomes (RR 1.16, 95% CI 1.07–1.26). Thirty-day mortality was lower with MIS (RR 0.63, 95% CI 0.49–0.80). No significant differences were observed for ICU stay (Mean Difference, MD –0.15 days, 95% CI −1.34 to 1.05) or rebleeding (RR 1.57, 95% CI 0.84–2.97). Severe adverse events were lower in MIS (RR 0.80, 95% CI 0.71–0.89). *Conclusions:* MIS may reduce mortality and improve functional outcomes in supratentorial ICH, particularly using stereotactic aspiration with thrombolytics. The benefit in deep hematomas remains uncertain. Early intervention and careful patient selection are essential. Further high-quality RCTs are warranted.

## 1. Introduction

Minimally invasive surgery (MIS) has emerged as a promising approach for evacuating spontaneous intracerebral hemorrhage (ICH), a condition historically associated with high mortality and disability [1]. Current management focuses largely on medical stabilization; blood pressure control, reversal of coagulopathy, and prevention of complications [2]. However, medical therapy does not address the mass effect or secondary injury from the hematoma [3]. Conventional craniotomy has long been investigated to relieve mass effect, yet large randomized trials, including STICH II, failed to show clear functional benefit except in select subgroups (e.g., lobar, superficial bleeds without intraventricular extension) [4]. Accordingly, guidelines restrict surgery to life-saving scenarios, leaving its role in functional recovery uncertain [2]. MIS techniques, including stereotactic aspiration with thrombolysis, endoscopic evacuation, and trans-sulcal parafascicular approaches, aim to remove hematomas while minimizing disruption to surrounding brain tissue [5]. Compared with craniotomy, MIS reduces perioperative morbidity and accelerates recovery [6,7]. The critical question, however, is whether MIS improves outcomes over standard medical management, the default therapy for most patients.

Recent large trials have reshaped this discussion. The MISTIE III trial, although neutral on its primary endpoint, suggested that the larger the hematoma reduction, the better the outcomes, rekindling interest in MIS [8]. The ENRICH trial further advanced the field by demonstrating improved 180-day utility-weighted modified Rankin Scale scores using MIS, mostly in lobar hematoma [9]. These gaps highlight the need for individualized treatment strategies and justify our updated systematic review and meta-analysis, incorporating recent RCTs and prospective propensity score–matched cohort studies comparing MIS with medical management alone in adults with spontaneous supratentorial ICH.

## 2. Methods

This study followed the Preferred Reporting Items for Systematic Reviews and Meta-Analyses (PRISMA) guidelines [10].

### 2.1. Search Strategy

A comprehensive search of PubMed, Web of Science, Scopus, and the Cochrane Library was performed on 8 September 2025. We used a combination of controlled vocabulary and free-text terms related to the population, intervention, and comparator of interest. The detailed search terms and database-specific results are provided in Table S1. In addition to the database searches, we manually screened the reference lists of all eligible articles, as well as relevant systematic reviews and meta-analyses to identify additional studies. Retrieved studies were imported to Endnote to detect and remove duplicates, then imported into Rayyan software for screening. Two reviewers (MMA & MSA) independently screened titles and abstracts for eligibility, followed by full-text review. Any disagreements were resolved by discussion and, when necessary, adjudicated by a third reviewer (AIQ).

### 2.2. Eligibility Criteria

We applied explicit eligibility criteria based on a predefined PICO framework and study design filters to ensure transparency and reproducibility. We included studies enrolling adult patients with spontaneous supratentorial ICH, whether deep or lobar, without restrictions on hematoma volume or baseline Glasgow Coma Scale. Eligible interventions consisted of any MIS evacuation technique, including stereotactic aspiration with or without thrombolysis, endoscopic evacuation, tubular retractor–assisted or transsulcal parafascicular approaches, and robot-assisted systems. The comparator was medical management alone, defined as guideline-based therapy encompassing blood pressure optimization, reversal of coagulopathy, intracranial pressure control, and supportive neurocritical care [2]. Studies were required to report functional outcomes, such as the modified Rankin Scale (mRS), or mortality specific to MIS-treated cases. Only RCTs and prospectively designed propensity score–matched cohorts were included.

We excluded studies evaluating conventional craniotomy, those focused on brainstem hemorrhage, retrospective cohorts, unmatched prospective observational studies, small feasibility or pilot trials, and any study lacking outcome data relevant to MIS. The inclusion of diverse MIS techniques, surgical timing windows, and follow-up durations was intentional, as the field is highly heterogeneous and rapidly evolving. Incorporating this variability allowed us to compare performance across techniques and identify patterns that may guide future standardization and trial design. Conversely, retrospective cohorts were excluded to minimize confounding and selection bias, which are particularly problematic in ICH research where baseline severity strongly determines outcomes. By restricting the evidence base to RCTs and well-matched prospective cohorts, we aimed to ensure that our conclusions regarding the comparative effectiveness of MIS are grounded in the highest-quality and most methodologically robust data available.

### 2.3. Data Extraction, Risk of Bias, and Grading of Evidence

Data extraction was performed independently by two authors using a pre-designed Excel spreadsheet, and all entries were double-checked for accuracy. Conversions were applied where necessary to ensure consistent reporting, including transformation of data into event-to-total counts and mean ± standard deviation. Extracted variables included study characteristics (first author name, year of publication, study design and setting, study duration, trial name or registration number, and eligibility criteria), sample size (MIS vs. medical management [MM]), type of MIS technique, duration of follow-up (months), reported outcomes, and main findings. Baseline patient data were also recorded, including mean age (years), sex distribution (% male), hematoma volume (mL, mean ± SD), hematoma location (deep, lobar, or mixed), time from onset to randomization or treatment (mean ± SD), baseline GCS, presence of intraventricular hemorrhage (IVH, %), and comorbidities.

Risk of bias was assessed separately for randomized and non-randomized studies. For randomized controlled trials, we used the Cochrane Risk of Bias 2.0 tool [11]. For prospective propensity score–matched cohort studies, we applied the Risk Of Bias In Non-randomized Studies of Interventions (ROBINS-I) tool [12]. Two reviewers performed the risk of bias assessment independently, and disagreements were resolved through consensus or consultation with a third reviewer. The certainty of evidence for key outcomes (Good functional outcomes and 30-day mortality) was evaluated using the GRADE (Grading of Recommendations, Assessment, Development, and Evaluation) approach. Each outcome was assessed across the domains of risk of bias, inconsistency, indirectness, imprecision, and publication bias. Evidence certainty was classified as high, moderate, low, or very low.

### 2.4. Statistical Analysis

We performed a meta-analysis using Review Manager (RevMan) version 5.4. The primary outcomes were functional outcomes and mortality, while secondary outcomes included severe adverse events, rebleeding, and intensive care unit (ICU) length of stay. For dichotomous variables, we calculated risk ratios (RRs) with corresponding 95% confidence intervals (CIs), and for continuous variables, we used mean differences (MDs) with 95% CIs. Based on the recommendations of the Cochrane Handbook for Systematic Reviews of Interventions, a fixed-effects model (Mantel–Haenszel method) was used if statistical heterogeneity was considered low (I^2^ < 50% and *p* > 0.10). Otherwise, if heterogeneity was detected, a random-effects model, applying the DerSimonian and Laird method, was adopted. Statistical heterogeneity was measured using the I^2^ statistic [13]. Pre-specified subgroup analyses were conducted for functional outcomes based on (1) study design (RCTs vs. cohort studies), (2) ICH location (deep vs. mixed), (3) follow-up duration, and (4) type of MIS. Exploratory subgrouping was also considered according to (1) time from onset to surgery (<24, <48, or <72 h) and (2) publication period (before 2010, 2010–2020, and after 2020) to assess the potential influence of advances in technique and perioperative timing on clinical outcomes. For mortality, we conducted two separate analyses: one for 30-day mortality and one for mortality at the last available follow-up. To assess the possibility of publication bias, we generated funnel plots for the two primary analyses (functional outcomes and mortality) in cases where the number of included studies was sufficient to allow meaningful interpretation.

## 3. Results

### 3.1. Search Results

**Figure 1:**
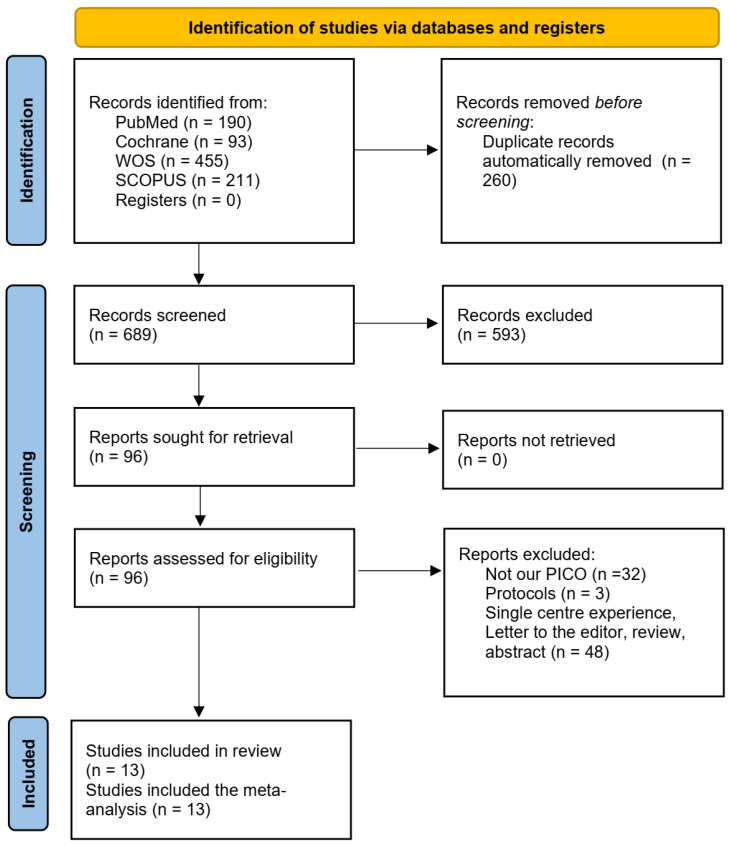
The PRISMA flowchart.

The database search yielded a total of 949 records. After removing duplicates, 689 records remained for screening. Based on the title and abstract review, 96 full-text articles were assessed for eligibility according to the inclusion criteria. Ultimately, 13 studies were included [8,9,14,15,16,17,18,19,20,21,22,23,24]. The PRISMA flowchart is shown in Figure 1.

### 3.2. Study Characteristics

A total of 13 studies published between 1989 and 2025 were included, encompassing both RCTs [8,9,14,17,18,19,20,21,22,23,24] and prospective matched cohort studies [15,16]. Together, these studies reported outcomes for 1503 patients treated with MIS and 1401 patients treated with conventional MM. The sample size of individual studies ranged from 35 to 499 participants. A summary of included studies is shown in Table 1. The mean age of participants across studies ranged from the mid-50 s to late-60 s, and most studies reported a male predominance. Hematoma volumes at baseline varied widely, with means generally between 30 and 60 mL. Studies differed in whether they enrolled primarily deep ICH (e.g., basal ganglia, thalamus) or mixed cohorts. Time from symptom onset to randomization or treatment initiation was typically within 24 h, though some studies extended inclusion up to 72 h. Baseline severity was commonly assessed using the GCS and the National Institutes of Health Stroke Scale (NIHSS), with reported GCS scores at presentation usually in the range of 7–12. Intraventricular hemorrhage (IVH) at baseline was variably reported but present in a substantial proportion of patients. Baseline characteristics of included studies are demonstrated in Table 2.

Outcome measures were heterogeneous. Most studies assessed functional recovery using mRS, but thresholds for defining a “good outcome” differed. Sun (2025) [15] and Hattori (2004) [22] defined good outcome as mRS ≤ 2, whereas most other trials defined it as mRS ≤ 3. Several studies reported outcomes at multiple follow-up intervals: 90 days, 180 days, and in some cases 1 year. Arthur (2025) [14] and Pradilla (2024) [9] incorporated utility-weighted mRS to quantify disability, while Deng (2022) [17] used both NIHSS scores after treatment and the Glasgow Outcome Scale (GOS) at 6 months. Mortality was assessed in nearly all studies, though reporting intervals differed. Some studies reported early mortality (30 days), while others extended follow-up to 180 days or 1 year.

### 3.3. Quality Assessment and Grading of Evidence

**Figure 2:**
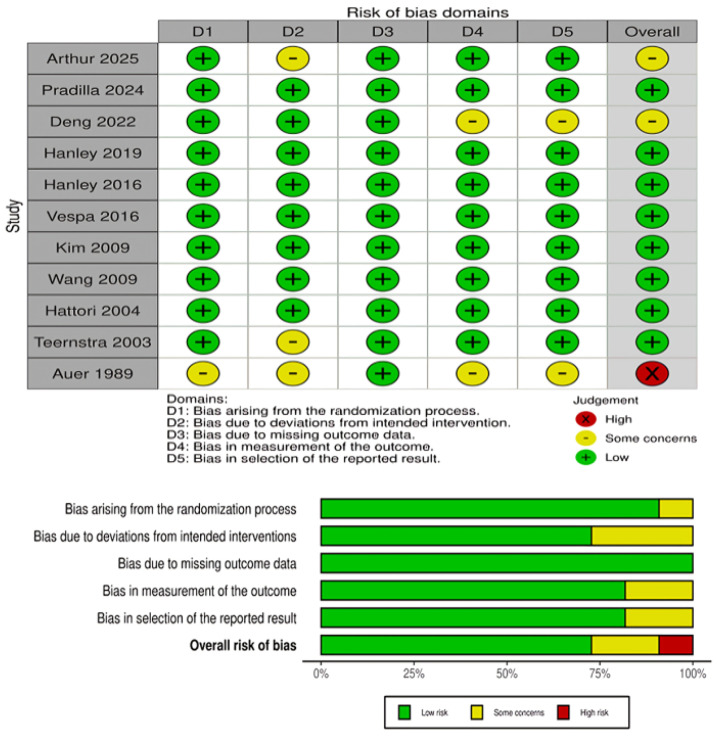
Risk of bias assessment for RCTs using the Cochrane RoB-2. The panels above show domain-level judgments for individual trials, while the panels below present the aggregate proportion of studies rated as low risk, some concerns, or high risk across each domain [8,9,14,17,18,19,20,21,22,23,24].

**Figure 3:**
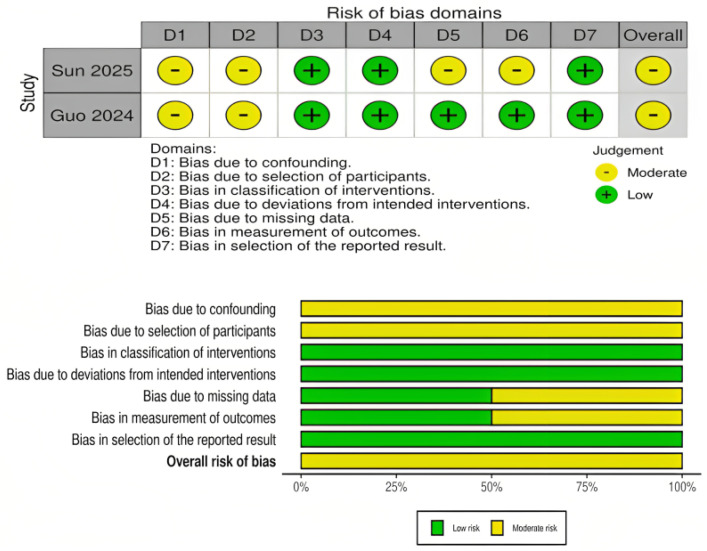
Risk of bias assessment for propensity score–matched cohort studies using the ROBINS-I tool. The panels above display domain-level judgments for individual studies, and the panels below summarize the aggregate proportion of studies rated as low, moderate, or serious risk across each domain [15,16].

The risk of bias assessment for RCTs is presented in Figure 2. Most RCTs demonstrated a low risk of bias across the majority of domains, particularly in outcome measurement and reporting. However, several trials showed “some concerns” in domains related to deviations from the intended interventions, missing outcome data, and selection of the reported result. One early trial (Auer, 1989) [24] was judged to have a high overall risk of bias due to deficiencies in the randomization process and selective reporting. Overall, the RCT evidence base was judged to be of moderate-to-high quality. For the observational cohort studies, risk of bias was assessed using the ROBINS-I tool and is presented in Figure 3. Both included studies (Sun 2025 and Guo 2024) [15,16] were judged to have a moderate overall risk of bias. Key concerns included residual confounding, potential selection bias, minor uncertainty in intervention classification, low rates of missing outcome data, and the lack of explicit blinding in outcome assessment. Other ROBINS-I domains were low risk.

According to GRADE assessment (Table S2), the certainty of evidence for good functional outcomes was rated as low. Although a significant effect favoring MIS was observed, heterogeneity across RCTs and variability in outcome definitions lowered the certainty. For mortality outcomes, the certainty of evidence was rated as moderate. 30-day mortality consistently favored MIS, with minimal heterogeneity and precise estimates. However, the overall certainty was downgraded by one level due to some concerns regarding risk of bias and the inclusion of non-randomized studies.

### 3.4. Meta-Analysis

#### 3.4.1. Achieving Good Functional Outcomes

**Figure 4:**
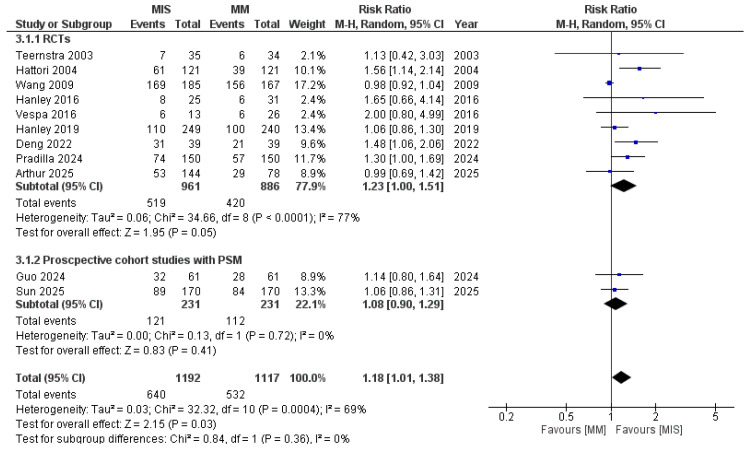
Forest plot of good functional outcomes comparing minimally invasive surgery (MIS) with medical management (MM), subgrouped according to study design [8,9,14,15,16,17,18,19,21,22,23].

In the pooled analysis of 11 studies, MIS was associated with significantly better functional outcomes compared with MM (RR 1.18, 95% CI 1.01–1.38, *p* = 0.03) Figure 4. However, statistical heterogeneity was high (I^2^ = 69%, *p* < 0.0001), indicating variability across included studies. Given the variability in definitions of favorable functional outcome, we performed a sensitivity analysis excluding studies that defined good outcome as mRS ≤ 2 (Sun 2025, Hattori 2004). The pooled effect estimate remained statistically significant (RR 1.10, 95% CI 1.01–1.19, *p* = 0.03) with slightly reduced heterogeneity (I^2^ = 63%) (Figure S1). This indicates that the overall benefit of MIS on functional outcomes is robust and not solely driven by differences in outcome definition.

Visual inspection of the funnel plot (Figure S2) suggested slight asymmetry. However, Egger’s regression test did not show statistically significant evidence of small-study effects (intercept = 0.81, *p* = 0.16), indicating that publication bias is unlikely to have substantially influenced our findings.

Pre-specified subgroup analyses were performed to investigate the sources and help decision making. When stratified by study designs, RCTs showed a significant effect in favor of MIS (RR 1.23, 95% CI 1.00–1.51; I^2^ = 77%); however, the difference is borderline significant (*p* = 0.05). Propensity score–matched cohort studies did not demonstrate a significant benefit (RR 1.08, 95% CI 0.90–1.29; I^2^ = 0%) (Figure 4).

**Figure 5:**
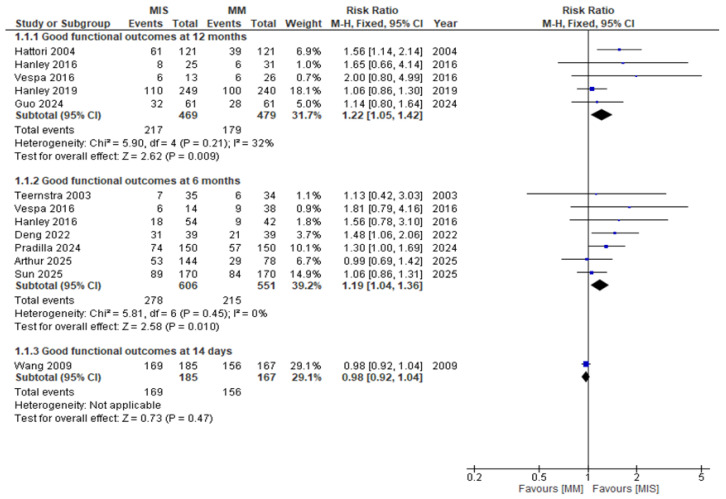
Forest plot of good functional outcomes comparing minimally invasive surgery (MIS) with medical management (MM), stratified by follow-up duration (12 months, 6 months, and 14 days) [8,9,14,15,16,17,18,19,22,23].

When studies were stratified according to follow-up duration, the heterogeneity observed in the pooled analysis resolved (Figure 5). At 12 months, patients undergoing MIS had significantly higher rates of good functional outcomes compared with those receiving MM (RR 1.22, 95% CI 1.05–1.42; *p* = 0.009; I^2^ = 32%). Similarly, at 6 months, MIS demonstrated a significant advantage (RR 1.19, 95% CI 1.04–1.36; *p* = 0.01; I^2^ = 0%). In contrast, early outcomes assessed at 14 days showed no difference between groups (RR 0.98, 95% CI 0.92–1.04; *p* = 0.47).

**Figure 6:**
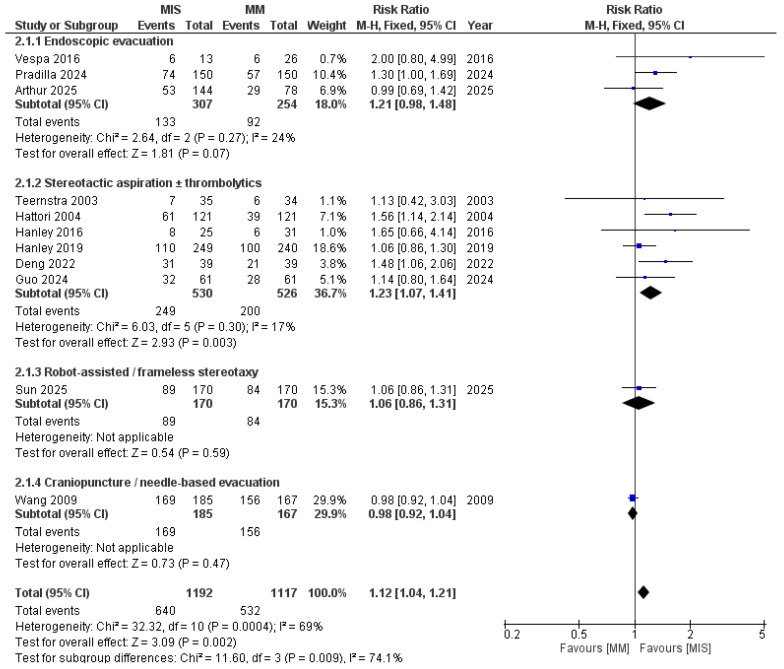
Forest plot of good functional outcomes comparing minimally invasive surgery (MIS) with medical management (MM), subgrouped by MIS technique. Studies using stereotactic aspiration and thrombolytics showed more favorable outcomes [8,9,14,15,16,17,18,19,21,22,23].

As shown in Figure 6, when studies were subgrouped by MIS technique, heterogeneity was also resolved. MIS showed a statistically significant advantage over MM in studies using stereotactic aspiration with local thrombolytics (RR = 1.23, 95% CI 1.07–1.41; I^2^ = 17%; *p* = 0.003). In endoscopic evacuation studies, the difference favored MIS but did not reach statistical significance (RR = 1.21, 95% CI 0.98–1.48; I^2^ = 24%; *p* = 0.07). In craniopuncture and robot-assisted MIS, each represented by a single study, no significant difference was observed.

**Figure 7:**
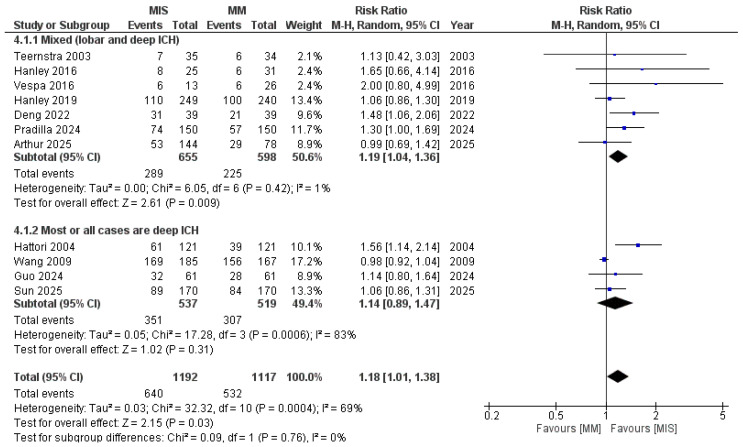
Forest plot of good functional outcomes comparing minimally invasive surgery (MIS) with medical management (MM), subgrouped by ICH location. Studies including mixed locations demonstrated a significant benefit of MIS, whereas studies limited to deep ICH did not show a significant difference [8,9,14,15,16,17,18,19,21,22,23].

When subgrouped based on the location of ICH (Figure 7), studies including mixed locations demonstrated a significant difference in favor of MIS (RR 1.19, 95% CI 1.04–1.36; I^2^ = 1%; *p* = 0.009). In contrast, the deep ICH subgroup showed no significant difference (RR 1.14, 95% CI 0.89–1.47; *p* = 0.31), with substantial heterogeneity observed. After excluding the study by Hattori et al., heterogeneity was reduced (I^2^ = 16%); however, the results remained non-significant (*p* = 0.99) (Figure S3).

When stratified by time from onset to surgery (Figure S4), studies performing MIS within 24 h demonstrated a significant improvement in functional outcomes compared with medical management (RR = 1.16, 95% CI 1.07–1.26, *p* = 0.0006), though with high heterogeneity (I^2^ = 89%). In contrast, studies with interventions performed within 48 h (RR = 1.12, 95% CI 0.92–1.36, *p* = 0.24, I^2^ = 20%) and within 72 h (RR = 1.06, 95% CI 0.83–1.36, *p* = 0.64, I^2^ = 0%) showed no statistically significant differences.

When stratified by year of publication (Figure S5), more recent studies published after 2020 continued to demonstrate a significant advantage for MIS (RR = 1.16, 95% CI 1.02–1.31, *p* = 0.03, I^2^ = 6%), whereas those published between 2010 and 2020 (RR = 1.16, 95% CI 0.92–1.36, *p* = 0.24, I^2^ = 20%) and before 2010 (RR = 1.09, 95% CI 1.00–1.19, *p* = 0.05, I^2^ = 89%) showed smaller or borderline effects. No significant subgroup differences were detected (*p* = 0.77), indicating the consistency of MIS benefit across different publication periods.

#### 3.4.2. Mortality

**Figure 8:**
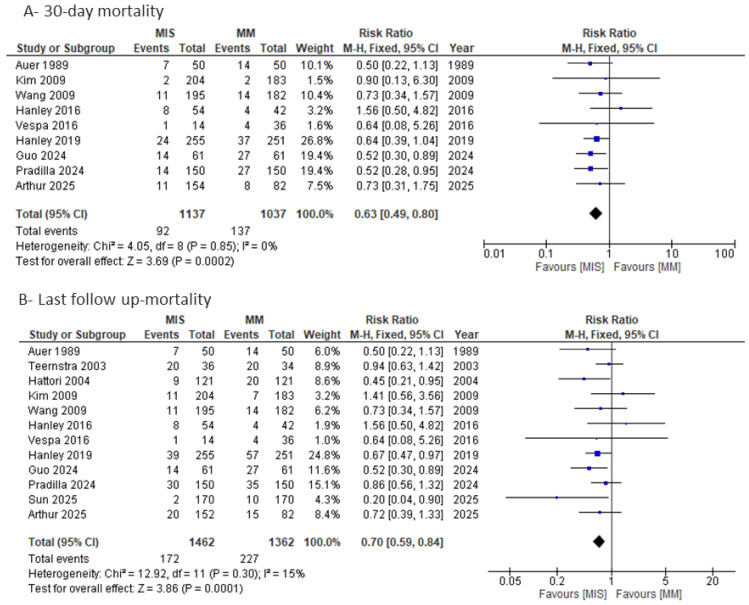
Forest plots of mortality comparing minimally invasive surgery (MIS) with medical management (MM): (**A**) 30-day mortality and (**B**) last follow-up mortality [8,9,14,15,16,17,18,19,20,21,22,23,24].

As shown in Figure 8A, 30-day mortality was significantly lower in the MIS group compared with MM (RR = 0.63, 95% CI 0.49–0.80; I^2^ = 0%; *p* = 0.0002). When pooling studies that reported mortality at varying follow-up periods, the analysis of last follow-up mortality (Figure 8B) also demonstrated a significant reduction in the MIS group (RR = 0.70, 95% CI 0.59–0.84; I^2^ = 15%; *p* = 0.0001). Assessment of publication bias using a funnel plot (Figure S6) revealed slight asymmetry on visual inspection; however, Egger’s regression test did not indicate significant small-study effects (*p* = 0.56).

#### 3.4.3. ICU Length of Stay, Risk of Rebleeding, and Severe Adverse Events

Regarding ICU length of stay, no significant difference was observed between the MIS and MM groups (MD = −0.15 days, 95% CI −1.34 to 1.05; *p* = 0.81). However, substantial heterogeneity was present (I^2^ = 84%) and was not resolved by a leave-one-out sensitivity analysis (Figure S7). Stratifying by MIS technique (Figure S8) completely resolved heterogeneity: stereotactic aspiration with local thrombolytics was associated with a significantly longer ICU stay compared with MM (MD = 0.85 days, 95% CI 0.55–1.15; I^2^ = 0%; *p* < 0.0001), whereas endoscopic MIS was associated with a significantly shorter ICU stay (MD = −2.11 days, 95% CI −3.27 to −0.95; I^2^ = 28%; *p* = 0.0004). However, the findings of these subgroups are not reliable due to limited number of studies in each subgroup.

The risk of rebleeding was comparable between the MIS and MM groups, though numerically higher in the MIS group without reaching statistical significance (RR = 1.57, 95% CI 0.84–2.97; I^2^ = 46%; *p* = 0.16) (Figure S9).

The risk of severe adverse events, such as rebleeding, infection, or neurological deterioration, was lower in MIS (RR = 0.80, 95% CI 0.71–0.89; I^2^ = 0%; *p* = 0.0001) (Figure S10).

## 4. Discussion

The international guidelines, including those from the AHA/ASA, ESO, and CSA, generally recommend medical management as the standard treatment for most patients with supratentorial ICH [2,25,26]. These recommendations support surgical intervention only for selected patients. MIS has emerged as a less traumatic alternative to conventional craniotomy, with potential to reduce perihematomal injury, although evidence for functional benefit remains inconsistent and appears dependent on technique, hematoma location, timing, and patient selection [2,27,28].

In this updated meta-analysis, MIS was associated with lower 30-day and longer-term mortality compared with medical management, while functional improvement was modest and primarily observed in studies using stereotactic aspiration with local thrombolytics. Other MIS approaches, including endoscopic and robot-assisted techniques, did not demonstrate consistent functional advantages. Notably, outcomes in deep hematomas, particularly basal ganglia or thalamic ICH, remain uncertain, highlighting the importance of careful patient selection.

Previous evidence syntheses have consistently supported a role for MIS in ICH management, but with different comparator groups and methodological approaches. Alkhiri et al. pooled only high-quality randomized trials and demonstrated that MIS was superior to non-MIS strategies (medical management or craniotomy) for both functional outcomes and survival [29]. However, combining craniotomy and medical therapy in the control group may have obscured the specific effect of MIS relative to medical treatment alone. More recently, network meta-analyses have provided broader insights by comparing multiple surgical modalities simultaneously. Tariq et al. reported that MIS reduced mortality compared with conservative care, while demonstrating similar outcomes to conventional surgery or burr hole drainage, with added advantages such as shorter operating times, fewer complications, and reduced ICU stays [30]. Likewise, Huan et al. synthesized 31 RCTs involving 6448 patients and found moderate-certainty evidence that surgical interventions overall improved functional outcomes and reduced mortality compared with conservative management. Within surgical subtypes, both endoscopic and minimally invasive puncture techniques were associated with particularly favorable outcomes [31]. Our study builds on these findings by focusing on the most clinically relevant comparison, MIS versus medical management, while excluding conventional craniotomy arms. This design isolates the incremental benefit of MIS over optimized medical care and incorporates more recent RCTs and prospective propensity-matched cohorts, thus reflecting both trial evidence and pragmatic patient populations.

As demonstrated in the current meta-analysis, MIS had a statistically significant advantage over medical management only when stereotactic aspiration was combined with local thrombolytics. There findings are consistent with real-world evidence [32,33,34]. While a few individual studies have suggested potential benefits in deep ICH [15,20,35], our pooled estimates did not support a clear functional advantage in these cases. These findings indicate that the benefit of MIS is not universal but appears technique-specific and dependent on hematoma location, with local fibrinolysis in lobar hemorrhages providing the clearest clinical effect.

Several baseline characteristics impact outcomes beyond the studied subgroups in this meta-analysis. Hematoma volume, presentation GCS level of consciousness, patient age, and presence of IVH can have an impact on the final outcomes. These variables could not be considered for subgrouping due to inconsistent baseline data. A recent prospective multicenter propensity-matched cohort by Guo et al. examined freehand, non-navigated MIS for deep supratentorial ICH (volume ≥ 20 mL) and provides several instructive signals that we could not fully explore in our pooled analyses [16]. Notably, the mortality reduction with MIS was concentrated among patients with larger hematoma volume (≥47 mL) and those with more severe presentations (GCS 3–8), whereas patients with smaller volumes or higher GCS derived less clear short-term survival benefit. The freehand MIS group achieved a mean hematoma clearance of ~33% with a mean residual volume ~36 mL, substantially larger residuals than the ≤15 mL target associated with benefit in MISTIE [8], which may help explain why functional gains were not observed despite improved short-term survival.

The present meta-analysis provides important clinical practice insights. For patients with non-deep supratentorial ICH who present early after symptom onset, stereotactic aspiration with adjunctive thrombolysis appears to confer functional benefit and should be considered, provided that the procedure is performed in centers with sufficient expertise and infrastructure. By contrast, in deep ICH, particularly basal ganglionic and thalamic hemorrhages, the evidence for functional benefit remains uncertain. In such cases, conservative management remains reasonable until higher-quality data are available, and MIS should be reserved for carefully selected patients after multidisciplinary evaluation.

### Strengths and Limitations

The strengths of this meta-analysis include the rigorous and updated inclusion of recent randomized controlled trials and prospective matched cohort studies, providing a contemporary and clinically relevant comparison of MIS versus medical management. Prespecified subgroup and sensitivity analyses based on technique, hematoma location, follow-up duration, time to surgery, and publication year also helped clarify sources of heterogeneity. However, several limitations remain. MIS encompassed heterogeneous techniques with variable operator expertise, procedural targets, and reporting standards. Outcome definitions and follow-up intervals differed across studies, and some subgroup analyses were limited by few contributing trials, reducing statistical power. The inclusion of nonrandomized cohorts introduces residual confounding, while the absence of individual patient data precluded robust adjustment for key modifiers such as hematoma volume, baseline GCS, and IVH burden. We attempted to mitigate these issues through prespecified analyses, transparent stratified reporting, and conservative GRADE downgrading, but findings should be interpreted cautiously.

High-quality RCTs regarding stereotactic aspiration with thrombolytics, particularly in deep ICH, need to be conducted. Trials should stratify patients by hematoma volume, location, GCS, IVH presence, and timing of intervention. Outcome measures should be standardized and longer follow-up periods should be used to inform clinical practice and update guidelines. Adding imaging-based selection criteria and cost-effectiveness analyses will further inform optimal patient management. The exploratory analysis in MISTIE III, which revealed that greater clot removal is correlated with better functional outcome, can be the basis for generating the next set of trials. Subsequent studies should pre-specify procedural performance endpoints (e.g., end-of-treatment residual clot ≤15 mL and/or ≥70% clearance) and report achievement rates and reasons for failure. They should also explicitly test timing windows (including very early intervention <12–24 h versus later windows) while meticulously tracking rebleeding and other safety endpoints. Finally, powering head-to-head comparisons of MIS modalities (stereotactic aspiration ± thrombolytics, endoscopic, freehand techniques, robot-assisted) to cloth differences in residual clot and patient outcomes is essential.

## 5. Conclusions

MIS, particularly stereotactic aspiration with local thrombolytics, may reduce mortality in patients with supratentorial ICH, while functional benefits are modest, technique-specific, and uncertain in deep hematomas. However, these findings must be interpreted within the context of substantial heterogeneity across interventions, mixed study designs, non-uniform mRS cutoffs, and differing follow-up intervals, all of which limit the generalizability of the pooled results. Signals suggesting potential advantages of earlier intervention and technique-specific benefits arise from non-uniform evidence and should therefore be viewed as exploratory. High-quality, targeted RCTs, and comparative studies with standardized timing, outcome definitions, and technique protocols are needed to clarify indications and strengthen guideline recommendations.

## Figures and Tables

**Table 1:** Summary of the included studies.

ID	Study Design and Settings	Study Duration	Trial Name: NCT	Eligibility Criteria	Sample Size (MIS/MM)	Type of MIS	Follow up (Months)	Outcomes	Main Findings
Sun 2025 [15]	Prospective multicenter cohort study (PSM)	2019–2023	N/A	Adults (18–80), supratentorial sICH <30 mL	170/170	Frame- less robot-guided SA-CT	3, 6, and 12 months	1-year independence, complications, mortality	Frameless robot-guided SA-CT for small supratentorial hematomas with contralateral hemiplegia appears safe, improves standing recovery, and lowers mortality
Arthur 2025 [14]	Multicenter RCT	2018–2023	MIND: NCT03342664	Adults (18–80 years), ICH (20–80 mL), NIHSS ≥ 6, and GCS 5–15	154/82	Endoscopic (Artemis Neuro Evacuation Device (Penumbra Inc., Alameda, CA, USA))	6 months	180-day mRS score and 30-day mortality	MIS within 72 h showed no significant benefit over medical management in reducing 30-day mortality or 180-day disability in supratentorial ICH
Guo 2024 [16]	Prospective multicenter cohort study (PSM)	2014–2016	N/A	Adults (18–80 years), basal ganglia ICH (≥20 mL)	61/61	Freehand aspiration + thrombolysis	1 month 3 months, and 12 months	mRS at 90 days and 1 year, 30-day mortality, complications, and costs	MIS lowered short-term mortality, without long-term functional benefit; benefit confined to large bleeds and low GCS, with higher costs
Pradilla 2024 [9]	Multicenter RCT	2016–2022	ENRICH: NCT02880878	Adults (18–80 years), ICH (30–80 mL), GCS 5–14, NIHSS ≥ 6, premorbid mRS 0–1, Tx < 24 h	150/150	Endoport (BrainPath; clot resection using Myriad suction/cutting device (Portage, MI, USA)	6 months	180-day mRS score and 30-day mortality	Minimally invasive hematoma evacuation within 24 h improved 180-day functional outcomes vs. medical management, mainly in lobar ICH
Deng 2022 [17]	Multicenter RCT	2018–2019	Registration No. 004510321208	ICH, GCS > 7, Tx < 12 h	39/39	Freehand aspiration + thrombolysis	6 months	Functional outcomes by NIHSS	MIS improves recovery, enhances neurological outcomes, and reduces complications in ICH
Hanley 2019 [8]	Multicenter RCT	2013–2017	MISTIE III: NCT01827046	Adults ≥ 18 years, spontaneous, non-traumatic, supratentorial ICH ≥ 30 mL	255/251	Image-guided MISTIE (+thrombolytics)	12 months	mRS score at 365 days	For moderate to large intracerebral hemorrhage, MISTIE did not improve the proportion of patients who achieved a good response 365 days after intracerebral hemorrhage
Hanley 2016 [18]	Multicenter RCT	2006–2013	MISTIE: NCT00224770	Adults (18–80 years), ICH ≥ 20 mL	54/42	Image-guided MISTIE (+thrombolytics)	6 months	mRS score at 180 days	MIS + rt-PA appears safe with an apparent advantage of better functional outcome at 180 days. Increased asymptomatic bleeding is a major cautionary finding
Vespa 2016 [19]	Multicenter RCT	2009–2012	ICES: NCT00224770	Adults (18–80 years), ICH ≥ 20 mL, GCS ≤ 14 or NIHSS ≥ 6, eligible for surgery within 48 h, pre-morbid mRS 0–1	14/36 control cohort from the MISTIE trial	Endoscopic aspiration	6 months and 12 months	mRS at 30, 90, 180, 270, and 365 days	Early CT-guided endoscopic surgery is safe, effective, and may improve recovery
Kim 2009 [20]	Single-center RCT	N/A	N/A	Age 30–80; GCS 13–15; unilateral motor weakness grade 0–2; SICH volume < 30 cm^3^; location: basal ganglia and thalamus	204/183	Stereotactic-guided hematoma evacuation + thrombolytics	6 months, with a mean follow-up period of 11.6 months	MBI, mRS, 30-day, and 6-month mortality rates	In patients with a small volume of SICH, stereotactic-guided evacuation improved functional recovery in activities in daily life than conservative treatment did
Wang 2009 [21]	Multicenter RCT	2003–2004	N/A	Adult (40–75); muscle strength grade 0–3; Tx < 72 h; GCS ≥ 9	195/182	Craniopuncture therapy using the YL-1 puncture needle	14 days and 3 months	ADL at 90 days; mortality ≤ 3 months; dependency (mRS > 2 or BI < 95) at 90 days	Minimally invasive craniopuncture is a safe, practical treatment that improves independent survival in small (25–40 mL) basal ganglion hemorrhage.
Hattori 2004 [22]	Multicenter RCT	1998–2000	N/A	Adults (35–85 years), Tx < 24 h, only neurological grades 2 and 3 were included	121/121	Stereotactic hematoma evacuation	12 months	Mortality and functional independence (mRS 0–2)	Multivariate analysis confirmed MIS as an independent predictor of better functional recovery
Teernstra 2003 [23]	Multicenter RCT	1996–1999	SICHPA: N/A	Age 45 years, GCS (2–10), volume > 10 mL, Tx ≤ 72 h.	36/34	Stereotactic-guided hematoma evacuation + thrombolytics	1 month 3 months, and 6 months	Mortality and functional outcomes	Stereotactic aspiration may improve prognosis in ICH
Auer 1989 [24]	Single-center RCT	1983–1986	N/A	Adult (30–80); hematoma > 10 cm^3^; neuro deficits/↓consciousness; fit for surgery; Tx ≤ 48 h; carotid angiography feasible	50/50	Endoscopic evacuation	6 months	Mortality and quality of survival at 6 months; early mortality ≤ 1 week; morbidity	Early endoscopic evacuation of subcortical spontaneous intracerebral hemorrhages is a safe and effective procedure that can reduce mortality and improve functional recovery in select patient subgroups

Abbreviations: RCT, randomized controlled trial; PSM, propensity score–matched; MIS, minimally invasive surgery; MM, medical management; sICH, spontaneous intracerebral hemorrhage; ICH, intracerebral hemorrhage; Tx, treatment; NCT, ClinicalTrials.gov identifier; CT, computed tomography; mRS, modified Rankin Scale; NIHSS, National Institutes of Health Stroke Scale; GCS, Glasgow Coma Scale; MBI, Modified Barthel Index; ADL, activities of daily living; BI, Barthel Index; N/A, not available.

**Table 2:** Baseline characteristics of study populations.

Study ID	Mean Age (Years)	Sex (% Male)	Hematoma Volume (mL), Mean ± SD	Location (Deep/Lobar/Mixed)	Time from Onset to Randomization (Hours), Mean ± SD	GCS at Baseline	IVH Present (%)	Comorbidities
Sun 2025 [15]	55.7 ± 11.5	36.8%	19.2 ± 11.4	Deep: 100%	N/A	MM: 11.75 ± 2.83 MIS: 11.34 ± 3.18	24.1%	HTN 70.3%, Diabetes: 70.3%
Arthur 2025 [14]	60 ± 14.8	63.1%	20–80 mL, (41 ± 19.3)	Mixed: deep (69.5%), lobar (30.5%)	<72, 21 ± 12.6	5–12 (55.1%), 13–15 (44.9%)	40.7%	CVD 21%, HTN 80.9%, DM 25.8%, anticoagulant 6.4%
Guo 2024 [16]	55.7 ± 11.1	77.9%	49.5 ± 27.3 mL	Deep (100%)	<24	8.7 ± 4.4	N/A	HTN 68%, DM 13.1%, ACS history 0.8%, anticoagulant 0.8%
Pradilla 2024 [9]	64 ± 11.9	50%	20–80 mL, 55.0 ± 24.4	Mixed: deep (30.7), lobar (69.3%)	<24, 16.3 ± 7.9	4–8 (18%), 9–14 (82%)	41.3%	CVD 82%, CNS disease 30%
Deng 2022 [17]	62.07 ± 3.86	61%	35.25 ± 7.03	Mixed	<12, 5.69 ± 0.83	7–15 (100%)	N/A	N/A
Hanley 2019 [8]	61.7 ± 3.2	61.1%	45.8 ± 4.1	61.5%	46.6 ± 4.4	13–15: 30.8% 9–12: 43.9% 3–8: 25.5%	N/A	HTN: 96.4% Diabetes: 27.9% CVD: 14.4%
Hanley 2016 [18]	60.9 ± 11.5	65.6%	46.0 ± 17.9	Mixed: Deep (34.4%) Lober (65.6%)	MIS: 1.2 ± 0.5	13–15: 35.4% 9–12: 33.3% 3–8: 35.4%	N/A	DM: 26.0% HTN: 86.5%
Vespa 2016 [19]	59 ± 6.6	65%	39.4 ± 12.9	Mixed: deep 75%, lobar 25%	<48, 24.7 ± 7.3	9.7 ± 2	N/A	HTN 92%, DM 28.6%
Kim 2009 [20]	65.8 ± 8.7	74.7%	23.1 ± 3.4104	N/A	N/A	All patients had a GCS score of ≥13	N/A	HTN: 91.2% DM: 56.3% Hypercholesterolemia: 63.3% Previous stroke: 4.4% Smoking: 77.0% Chronic alcoholism: 30.0%
Wang 2009 [21]	56.7 ± 9.5	62.6%	32.6 ± 51.0	Basal ganglia: 100%	7.6 ± 11.2	12.0 ± 2.1	18.6%	HTN 55.2%
Hattori 2004 [22]	60 ± 10.2	61.2%	48 ± 15.6	Deep: putamen 100%	<24	N/A	N/A	HTN 53.7%
Teernstra 2003 [23]	70 ± 10	58%	>10 mL, 71 ± 29	Mixed: lobar 67%, deep 33%	<72, 12.5	9.5 ± 2.8	32%	HTN 47%, DM 10%, CVD 22%, Stroke history 25%, anticoagulant use 33%
Auer 1989 [24]	59.0 ± 12.3	61%	>50 mL: 46 <50 mL: 54	Lobar: 45% Deep: 55%	N/A	N/A§	N/A	HTN 78%

Abbreviations: MIS, minimally invasive surgery; MM, medical management; SD, standard deviation; GCS, Glasgow Coma Scale; IVH, intraventricular hemorrhage; HTN, hypertension; DM, diabetes mellitus; CVD, cardiovascular disease; ACS, acute coronary syndrome; CNS, central nervous system; mL, milliliter; N/A, not available.

## Data Availability

The data utilized in this meta-analysis were extracted from previously published studies. All data sources are publicly available and can be accessed through journals or databases. No new data were generated or collected for this study.

## Supplementary Materials

The following supporting information can be downloaded at https://www.mdpi.com/article/10.3390/medicina61122216/s1, Figure S1: A sensitivity analysis excluding studies defining good outcome as mRS ≤ 2 (Sun 2025, Hattori 2004) confirmed the robustness of this finding (RR 1.10, 95% CI 1.01–1.19, *p* = 0.03), with slightly reduced heterogeneity (I^2^ = 63%); Figure S2: Funnel plot assessing publication bias of good functional outcomes; Figure S3: Sensitivity analysis of the deep ICH subgroup after excluding Hattori et al; Figure S4: Forest plot comparing good functional outcomes between minimally invasive surgery (MIS) and medical management (MM), subgrouped by time from onset to surgery (<24 h, <48 h, and <72 h); Figure S5: Forest plot comparing good functional outcomes between minimally invasive surgery (MIS) and medical management (MM), subgrouped by year of publication (after 2020, 2010–2020, and before 2010); Figure S6: Funnel plot assessing publication bias in last-follow up-mortality; Figure S7. Forest plot of ICU length of stay comparing minimally invasive surgery (MIS) with medical management (MM); Figure S8: Forest plot of ICU length of stay subgrouped by MIS technique; Figure S9: Forest plot of rebleeding risk comparing minimally invasive surgery (MIS) with medical management (MM); Figure S10: Forest plot of severe adverse events comparing minimally invasive surgery (MIS) with medical management (MM); Table S1: Search terms and results; Table S2: GRADE assessment of the two primary outcomes.
