# Supplementary material for: Minimally Invasive Surgery Versus Medical Management for Spontaneous Supratentorial Intracerebral Hemorrhage: An Updated Systematic Review and Meta-Analysis of Randomized and Propensity Score–Matched Studies
Source: Medicina (Kaunas). 2025 Dec 16;61(12):2216. doi: 10.3390/medicina61122216 (PMC12735331; doi:10.3390/medicina61122216)
Supplement: Supplementary file 1 [file medicina-61-02216-s001.zip › medicina-4009481-supplementary.pdf]

Table S1. Search terms and results.

Database	Date	Search strategy	No
PUBMED	September 8th, 2025	#1 "intracerebral hemorrhage" OR "intracerebral haemorrhage" OR "intracranial hemorrhage" OR "intracranial haemorrhage" OR "brain hemorrhage" OR "brain haemorrhage" OR ICH OR intracerebral hematoma OR "intracerebral haematoma" OR "cerebral hemorrhage"<br>#2 "minimally invasive surgical procedures" OR "minimally invasive" OR "minimally-invasive" OR "minimally invasive surgery" OR MIS OR MIPS OR "stereotactic aspiration" OR "stereotactic evacuation" OR "stereotactic catheter" OR "endoscopic evacuation" OR "endoscopic aspiration" OR "endoscopic haematoma evacuation" OR "endoscopic hematoma evacuation" OR "tubular retractor" OR "brainpath" OR "transsulcal" OR "stereotactic aspiration" OR "stereotactic" OR "robot-assisted" OR "catheter-based evacuation" OR "minimally invasive surgery" OR "minimally invasive hematoma evacuation" OR "stereotactic aspiration with rt-PA" OR MISTIE<br>#3 "medical management" OR "conservative treatment" OR "conservative management" OR "nonoperative" OR "non-surgical" OR "best medical therapy" OR "guideline-based care" OR "standard medical treatment" OR "standard care" OR "usual care"<br>Final searching string: #1 AND #2 AND #3	190
WOS	September 8th, 2025	#1 "intracerebral hemorrhage" OR "intracerebral haemorrhage" OR "intracranial hemorrhage" OR "intracranial haemorrhage" OR "brain hemorrhage" OR "brain haemorrhage" OR ICH OR intracerebral hematoma OR "intracerebral haematoma" OR "cerebral hemorrhage"<br>#2 "minimally invasive surgical procedures" OR "minimally invasive" OR "minimally-invasive" OR "minimally invasive surgery" OR MIS OR MIPS OR "stereotactic aspiration" OR "stereotactic evacuation" OR "stereotactic catheter" OR "endoscopic evacuation" OR "endoscopic aspiration" OR "endoscopic haematoma evacuation" OR "endoscopic hematoma evacuation" OR "tubular retractor" OR "brainpath" OR "transsulcal" OR "stereotactic aspiration" OR "stereotactic" OR "robot-assisted" OR "catheter-based evacuation" OR "minimally invasive surgery" OR "minimally invasive hematoma evacuation" OR "stereotactic aspiration with rt-PA" OR MISTIE<br>#3 "medical management" OR "conservative treatment" OR "conservative management" OR "nonoperative" OR "non-surgical" OR "best medical therapy" OR "guideline-based care" OR "standard medical treatment" OR "standard care" OR "usual care"<br>Final searching string: #1 AND #2 AND #3	455
SCOPUS	September 8th, 2025	#1 "intracerebral hemorrhage" OR "intracerebral haemorrhage" OR "intracranial hemorrhage" OR "intracranial haemorrhage" OR "brain hemorrhage" OR "brain haemorrhage" OR ICH OR intracerebral hematoma OR "intracerebral haematoma" OR "cerebral hemorrhage"	211

		#2 "minimally invasive surgical procedures" OR "minimally invasive" OR "minimally-invasive" OR "minimally invasive surgery" OR MIS OR MIPS OR "stereotactic aspiration" OR "stereotactic evacuation" OR "stereotactic catheter" OR "endoscopic evacuation" OR "endoscopic aspiration" OR "endoscopic haematoma evacuation" OR "endoscopic hematoma evacuation" OR "tubular retractor" OR "brainpath" OR "transsulcal" OR "stereotactic aspiration" OR "stereotactic" OR "robot-assisted" OR "catheter-based evacuation" OR "minimally invasive surgery" OR "minimally invasive hematoma evacuation" OR "stereotactic aspiration with rt-PA" OR MISTIE<br>#3 "medical management" OR "conservative treatment" OR "conservative management" OR "nonoperative" OR "non-surgical" OR "best medical therapy" OR "guideline-based care" OR "standard medical treatment" OR "standard care" OR "usual care"<br>Final searching string: #1 AND #2 AND #3	
The COCHRANE Library	September 8th, 2025	#1 "intracerebral hemorrhage" OR "intracerebral haemorrhage" OR "intracranial hemorrhage" OR "intracranial haemorrhage" OR "brain hemorrhage" OR "brain haemorrhage" OR ICH OR intracerebral hematoma OR "intracerebral haematoma" OR "cerebral hemorrhage"<br>#2 "minimally invasive surgical procedures" OR "minimally invasive" OR "minimally-invasive" OR "minimally invasive surgery" OR MIS OR MIPS OR "stereotactic aspiration" OR "stereotactic evacuation" OR "stereotactic catheter" OR "endoscopic evacuation" OR "endoscopic aspiration" OR "endoscopic haematoma evacuation" OR "endoscopic hematoma evacuation" OR "tubular retractor" OR "brainpath" OR "transsulcal" OR "stereotactic aspiration" OR "stereotactic" OR "robot-assisted" OR "catheter-based evacuation" OR "minimally invasive surgery" OR "minimally invasive hematoma evacuation" OR "stereotactic aspiration with rt-PA" OR MISTIE<br>#3 "medical management" OR "conservative treatment" OR "conservative management" OR "nonoperative" OR "non-surgical" OR "best medical therapy" OR "guideline-based care" OR "standard medical treatment" OR "standard care" OR "usual care"<br>Final searching string: #1 AND #2 AND #3	93

Table S2. GRADE assessment of the two primary outcomes.

Outcome	№ of Participants (studies)	Study Design	Relative Effect (95% CI)	Absolute Effect	Certainty of the Evidence (GRADE)
Good functional outcomes	1824 (11 studies: 9 RCTs, 2 PSM cohort)	RCTs and prospective PSM studies	RR 1.18 (1.01–1.38)	180 per 1000 with MM → 212 per 1000 with MIS (18 more per 1000; 2 to 68 more)	●●○○ Low (downgraded for risk of bias, inconsistency)
30-day mortality	1650 (9 studies: RCTs and PSM cohort)	RCTs and prospective PSM studies	RR 0.63 (0.49–0.80)	210 per 1000 with MM → 132 per 1000 with MIS (78 fewer per 1000; 107 to 42 fewer)	●●●○ Moderate (downgraded for some risk of bias)

Study or Subgroup	MIS Events	MIS Total	MM Events	MM Total	Weight	Risk Ratio M-H, Fixed, 95% CI	Year
**3.1.1 RCTs**							
Teernstra 2003	7	35	6	34	1.4%	1.13 [0.42, 3.03]	2003
Hattori 2004	61	121	39	121	0.0%	1.56 [1.14, 2.14]	2004
Wang 2009	169	185	156	167	38.6%	0.98 [0.92, 1.04]	2009
Hanley 2016	8	25	6	31	1.3%	1.65 [0.66, 4.14]	2016
Vespa 2016	6	13	6	26	0.9%	2.00 [0.80, 4.99]	2016
Hanley 2019	110	249	100	240	24.0%	1.06 [0.86, 1.30]	2019
Deng 2022	31	39	21	39	4.9%	1.48 [1.06, 2.06]	2022
Pradilla 2024	74	150	57	150	13.4%	1.30 [1.00, 1.69]	2024
Arthur 2025	53	144	29	78	8.9%	0.99 [0.69, 1.42]	2025
**Subtotal (95% CI)**		**840**		**765**	**93.4%**	**1.09 [1.01, 1.19]**	
Total events	458		381				
Heterogeneity: Chi² = 21.17, df = 7 (P = 0.004); I² = 67%							
Test for overall effect: Z = 2.12 (P = 0.03)							
**3.1.2 Proscpective cohort studies with PSM**							
Guo 2024	32	61	28	61	6.6%	1.14 [0.80, 1.64]	2024
Sun 2025	89	170	84	170	0.0%	1.06 [0.86, 1.31]	2025
**Subtotal (95% CI)**		**61**		**61**	**6.6%**	**1.14 [0.80, 1.64]**	
Total events	32		28				
Heterogeneity: Not applicable							
Test for overall effect: Z = 0.72 (P = 0.47)							
**Total (95% CI)**		**901**		**826**	**100.0%**	**1.10 [1.01, 1.19]**	
Total events	490		409				
Heterogeneity: Chi² = 21.82, df = 8 (P = 0.005); I² = 63%							
Test for overall effect: Z = 2.24 (P = 0.03)							
Test for subgroup differences: Chi² = 0.05, df = 1 (P = 0.82), I² = 0%							

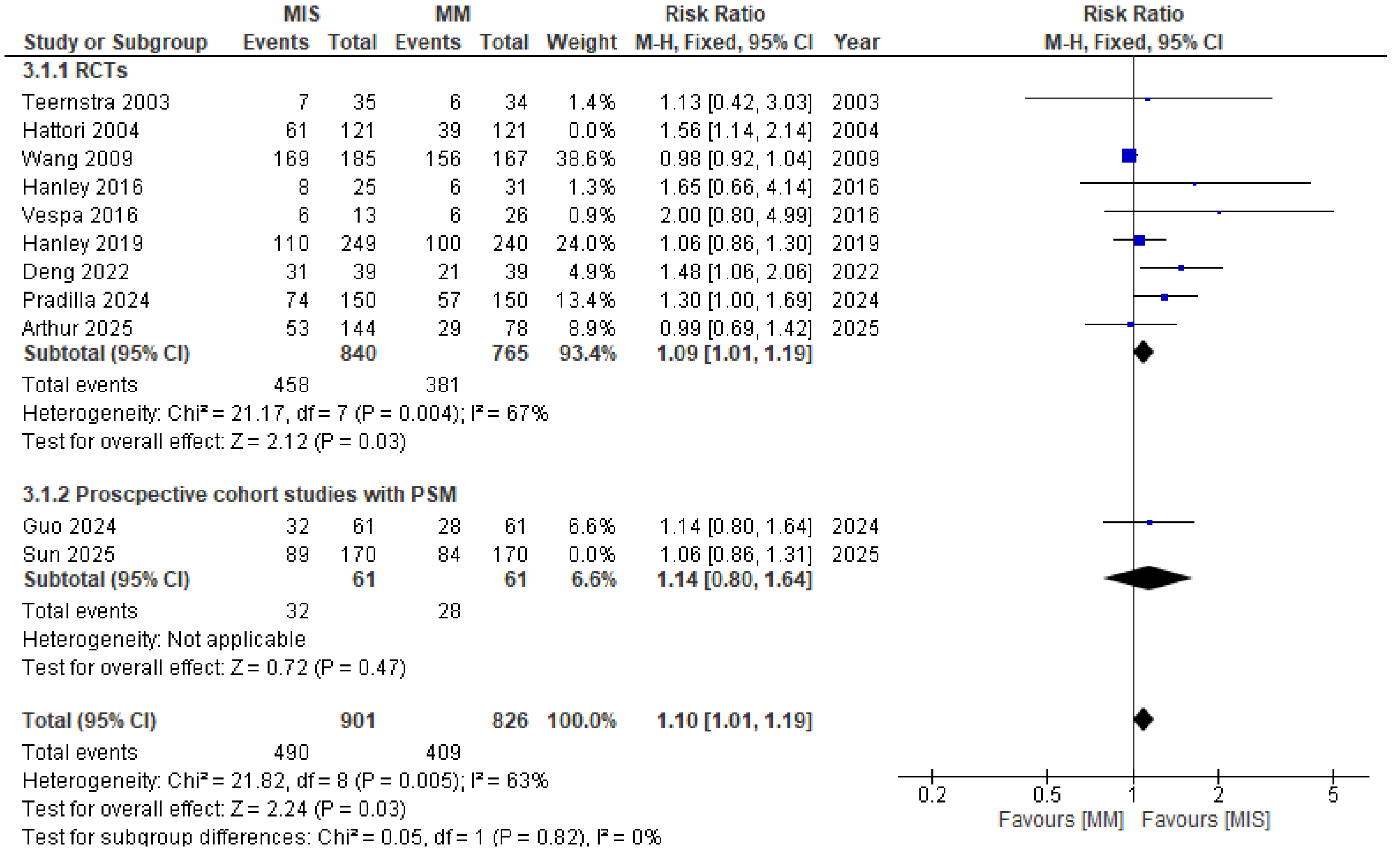

Figure S1. A sensitivity analysis excluding studies defining good outcome as mRS ≤2 (Sun 2025, Hattori 2004) confirmed the robustness of this finding (RR 1.10, 95% CI 1.01–1.19, p = 0.03), with slightly reduced heterogeneity (I² = 63%).

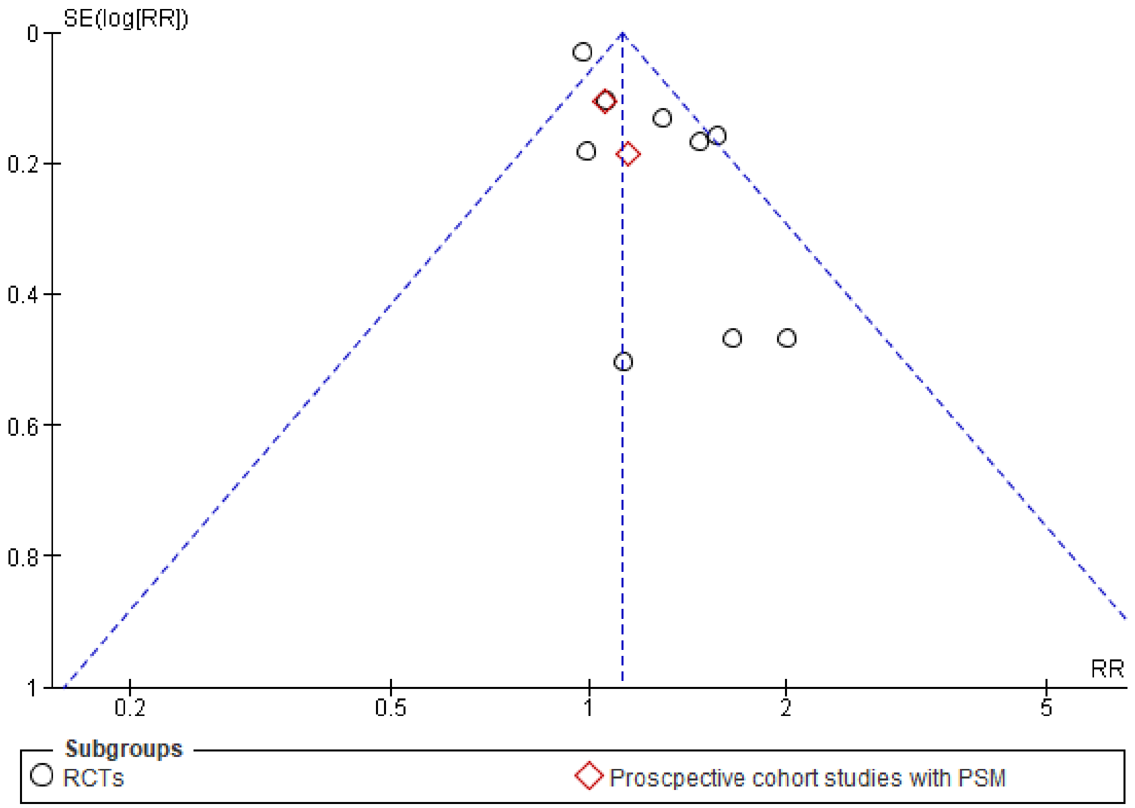

Figure S2. Funnel plot assessing publication bias of good functional outcomes. Each circle represents an individual randomized controlled trial, and each red diamond represents a prospective cohort study with propensity score matching. The vertical dashed line indicates the pooled risk ratio from the meta-analysis, and the diagonal lines represent pseudo 95% confidence limits. Visual inspection suggests slight asymmetry; however, Egger's regression test did not demonstrate statistically significant small-study effects ($p = 0.16$).

Study or Subgroup	MIS Events	MIS Total	MM Events	MM Total	Weight	Risk Ratio M-H, Random, 95% CI	Year
**4.1.1 Mixed (lobar and deep ICH)**							
Teernstra 2003	7	35	6	34	1.8%	1.13 [0.42, 3.03]	2003
Hanley 2016	8	25	6	31	2.0%	1.65 [0.66, 4.14]	2016
Vespa 2016	6	13	6	26	2.0%	2.00 [0.80, 4.99]	2016
Hanley 2019	110	249	100	240	15.5%	1.06 [0.86, 1.30]	2019
Deng 2022	31	39	21	39	9.9%	1.48 [1.06, 2.06]	2022
Pradilla 2024	74	150	57	150	12.7%	1.30 [1.00, 1.69]	2024
Arthur 2025	53	144	29	78	9.0%	0.99 [0.69, 1.42]	2025
**Subtotal (95% CI)**		**655**		**598**	**52.9%**	**1.19 [1.04, 1.36]**	
Total events	289		225				
Heterogeneity: Tau² = 0.00; Chi² = 6.05, df = 6 (P = 0.42); I² = 1%							
Test for overall effect: Z = 2.61 (P = 0.009)							
**4.1.2 Most or all cases are deep ICH**							
Hattori 2004	61	121	39	121	0.0%	1.56 [1.14, 2.14]	2004
Wang 2009	169	185	156	167	22.9%	0.98 [0.92, 1.04]	2009
Guo 2024	32	61	28	61	8.9%	1.14 [0.80, 1.64]	2024
Sun 2025	89	170	84	170	15.3%	1.06 [0.86, 1.31]	2025
**Subtotal (95% CI)**		**416**		**398**	**47.1%**	**1.00 [0.91, 1.09]**	
Total events	290		268				
Heterogeneity: Tau² = 0.00; Chi² = 2.37, df = 2 (P = 0.31); I² = 16%							
Test for overall effect: Z = 0.01 (P = 0.99)							
**Total (95% CI)**		**1071**		**996**	**100.0%**	**1.13 [0.99, 1.29]**	
Total events	579		493				
Heterogeneity: Tau² = 0.02; Chi² = 20.74, df = 9 (P = 0.01); I² = 57%							
Test for overall effect: Z = 1.76 (P = 0.08)							
Test for subgroup differences: Chi² = 4.69, df = 1 (P = 0.03), I² = 78.7%							

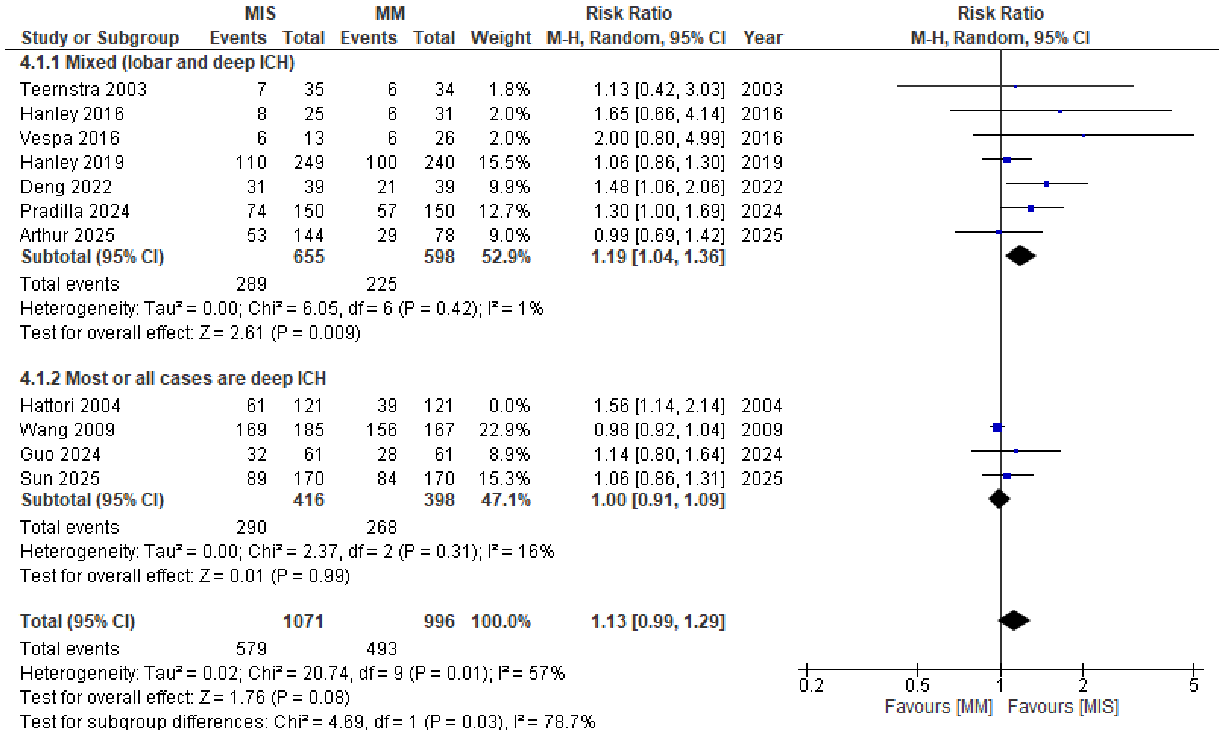

Figure S3. Sensitivity analysis of the deep ICH subgroup after excluding Hattori et al. Removal of this study reduced heterogeneity; however, the difference between MIS and medical management remained non-significant.

Study or Subgroup	MIS Events	MIS Total	MM Events	MM Total	Weight	Risk Ratio M-H, Fixed, 95% CI	Year
**10.1.1 <24 hours**							
Hattori 2004	61	121	39	121	7.9%	1.56 [1.14, 2.14]	2004
Wang 2009	169	185	156	167	33.3%	0.98 [0.92, 1.04]	2009
Deng 2022	31	39	21	39	4.3%	1.48 [1.06, 2.06]	2022
Guo 2024	32	61	28	61	5.7%	1.14 [0.80, 1.64]	2024
Pradilla 2024	74	150	57	150	11.6%	1.30 [1.00, 1.69]	2024
**Subtotal (95% CI)**		**556**		**538**	**62.8%**	**1.16 [1.07, 1.26]**	
Total events	367		301				
Heterogeneity: Chi² = 37.42, df = 4 (P < 0.00001); I² = 89%							
Test for overall effect: Z = 3.41 (P = 0.0006)							
**10.1.2 <48 hours**							
Hanley 2016	8	25	6	31	1.1%	1.65 [0.66, 4.14]	2016
Vespa 2016	6	13	6	26	0.8%	2.00 [0.80, 4.99]	2016
Hanley 2019	110	249	100	240	20.7%	1.06 [0.86, 1.30]	2019
**Subtotal (95% CI)**		**287**		**297**	**22.6%**	**1.12 [0.92, 1.36]**	
Total events	124		112				
Heterogeneity: Chi² = 2.51, df = 2 (P = 0.28); I² = 20%							
Test for overall effect: Z = 1.16 (P = 0.24)							
**10.1.3 <72 hours**							
Teernstra 2003	7	35	6	34	1.2%	1.13 [0.42, 3.03]	2003
Arthur 2025	53	144	29	78	7.6%	0.99 [0.69, 1.42]	2025
Sun 2025	32	61	28	61	5.7%	1.14 [0.80, 1.64]	2025
**Subtotal (95% CI)**		**240**		**173**	**14.6%**	**1.06 [0.83, 1.36]**	
Total events	92		63				
Heterogeneity: Chi² = 0.32, df = 2 (P = 0.85); I² = 0%							
Test for overall effect: Z = 0.47 (P = 0.64)							
**Total (95% CI)**		**1083**		**1008**	**100.0%**	**1.14 [1.05, 1.23]**	
Total events	583		476				
Heterogeneity: Chi² = 34.84, df = 10 (P = 0.0001); I² = 71%							
Test for overall effect: Z = 3.23 (P = 0.001)							
Test for subgroup differences: Chi² = 0.48, df = 2 (P = 0.79), I² = 0%							

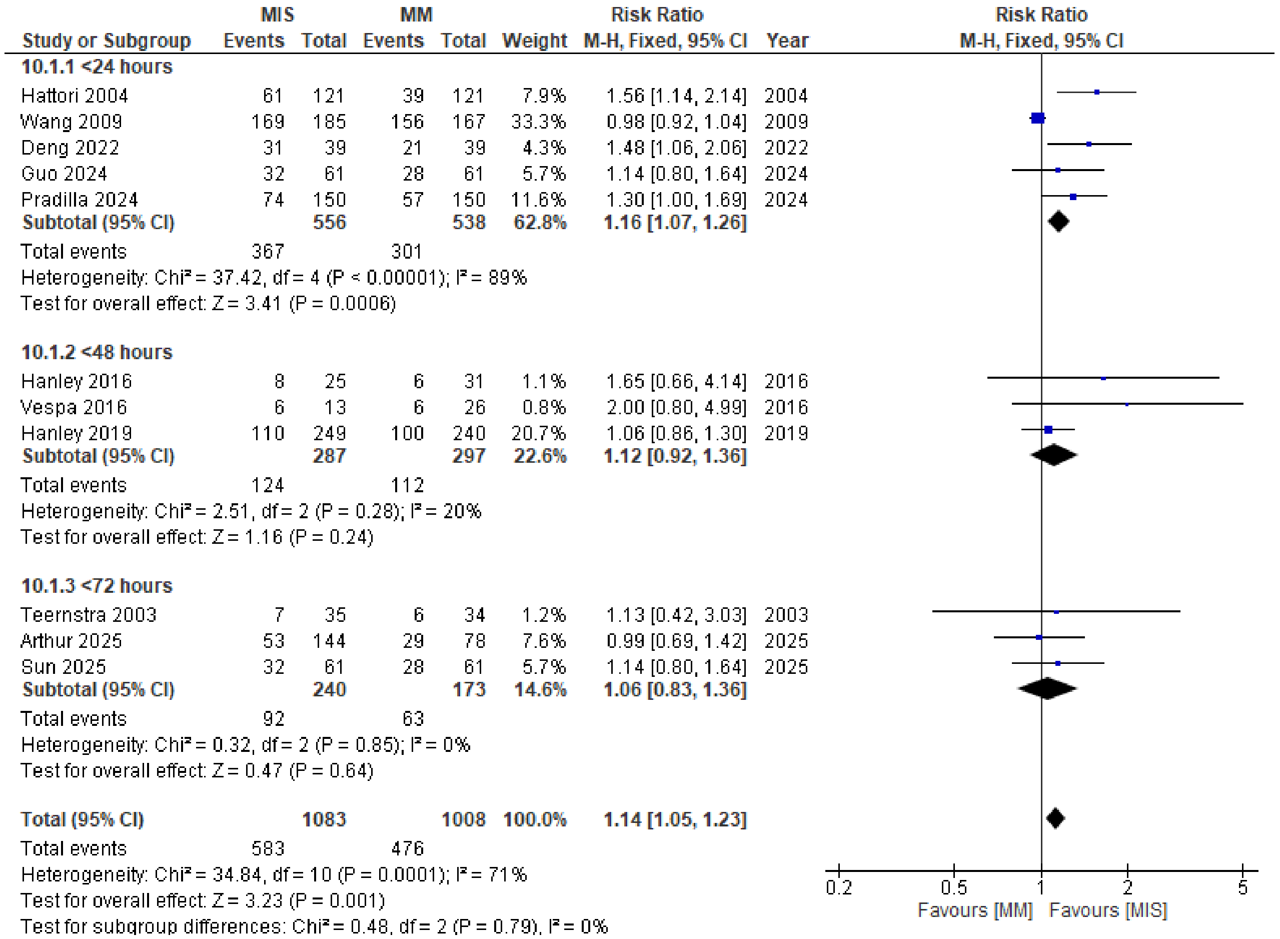

Figure S4. Forest plot comparing good functional outcomes between minimally invasive surgery (MIS) and medical management (MM), subgrouped by time from onset to surgery (<24 hours, <48 hours, and <72 hours).

Study or Subgroup	MIS Events	MIS Total	MM Events	MM Total	Weight	Risk Ratio M-H, Fixed, 95% CI	Year
**11.1.1 After 2020**							
Deng 2022	31	39	21	39	3.8%	1.48 [1.06, 2.06]	2022
Guo 2024	32	61	28	61	5.1%	1.14 [0.80, 1.64]	2024
Pradilla 2024	74	150	57	150	10.4%	1.30 [1.00, 1.69]	2024
Arthur 2025	53	144	29	78	6.9%	0.99 [0.69, 1.42]	2025
Sun 2025	89	170	84	170	15.3%	1.06 [0.86, 1.31]	2025
**Subtotal (95% CI)**		**564**		**498**	**41.5%**	**1.16 [1.02, 1.31]**	
Total events	279		219				
Heterogeneity: Chi² = 4.24, df = 4 (P = 0.38); I² = 6%							
Test for overall effect: Z = 2.22 (P = 0.03)							
**11.1.2 2010 to 2020**							
Vespa 2016	6	13	6	26	0.7%	2.00 [0.80, 4.99]	2016
Hanley 2016	8	25	6	31	1.0%	1.65 [0.66, 4.14]	2016
Hanley 2019	110	249	100	240	18.6%	1.06 [0.86, 1.30]	2019
**Subtotal (95% CI)**		**287**		**297**	**20.3%**	**1.12 [0.92, 1.36]**	
Total events	124		112				
Heterogeneity: Chi² = 2.51, df = 2 (P = 0.28); I² = 20%							
Test for overall effect: Z = 1.16 (P = 0.24)							
**11.1.3 Before 2010**							
Teernstra 2003	7	35	6	34	1.1%	1.13 [0.42, 3.03]	2003
Hattori 2004	61	121	39	121	7.1%	1.56 [1.14, 2.14]	2004
Wang 2009	169	185	156	167	29.9%	0.98 [0.92, 1.04]	2009
**Subtotal (95% CI)**		**341**		**322**	**38.2%**	**1.09 [1.00, 1.19]**	
Total events	237		201				
Heterogeneity: Chi² = 18.06, df = 2 (P = 0.0001); I² = 89%							
Test for overall effect: Z = 1.96 (P = 0.05)							
**Total (95% CI)**		**1192**		**1117**	**100.0%**	**1.12 [1.04, 1.21]**	
Total events	640		532				
Heterogeneity: Chi² = 32.32, df = 10 (P = 0.0004); I² = 69%							
Test for overall effect: Z = 3.09 (P = 0.002)							
Test for subgroup differences: Chi² = 0.54, df = 2 (P = 0.77), I² = 0%							

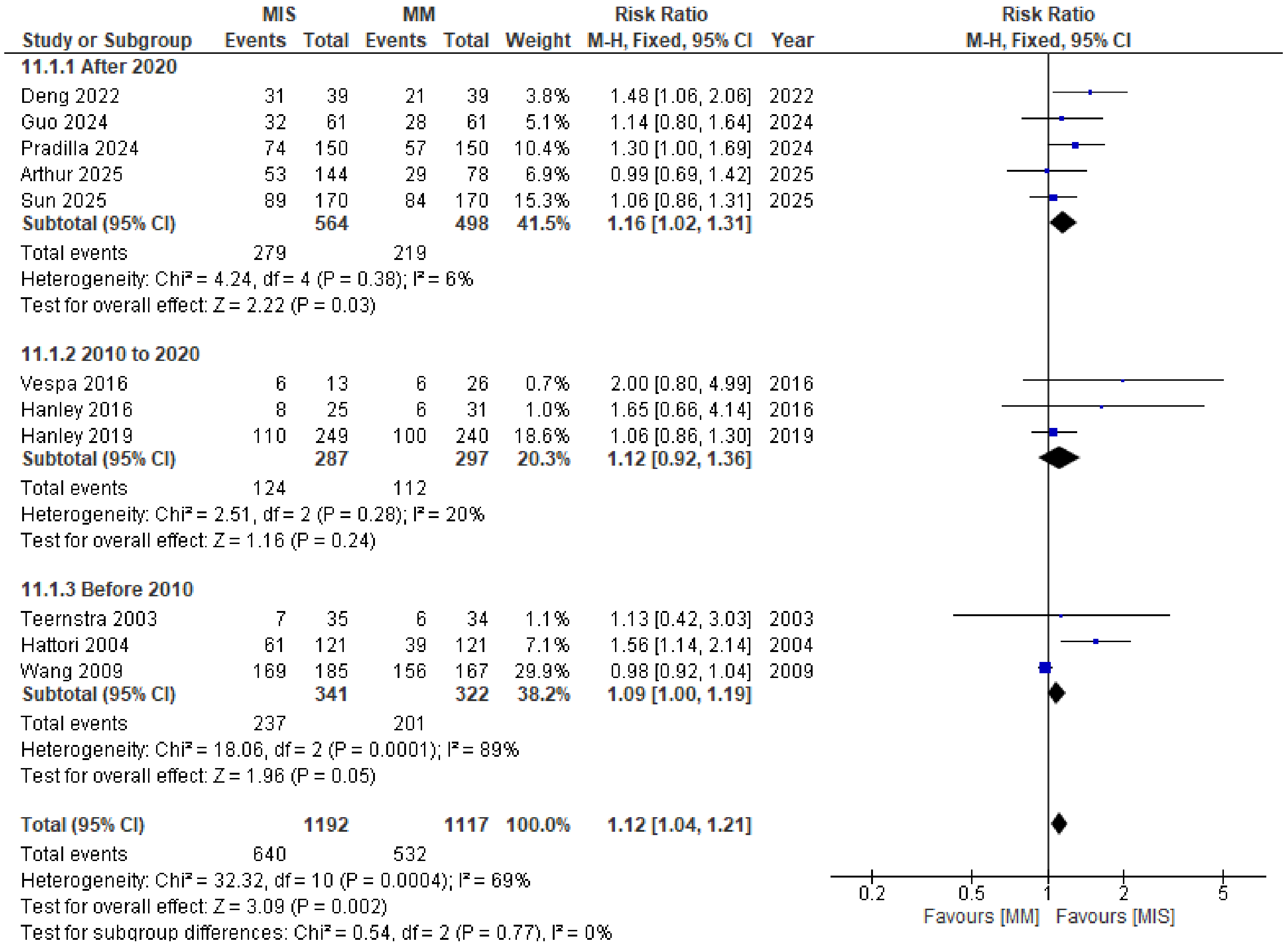

Figure S5. Forest plot comparing good functional outcomes between minimally invasive surgery (MIS) and medical management (MM), subgrouped by year of publication (after 2020, 2010–2020, and before 2010).

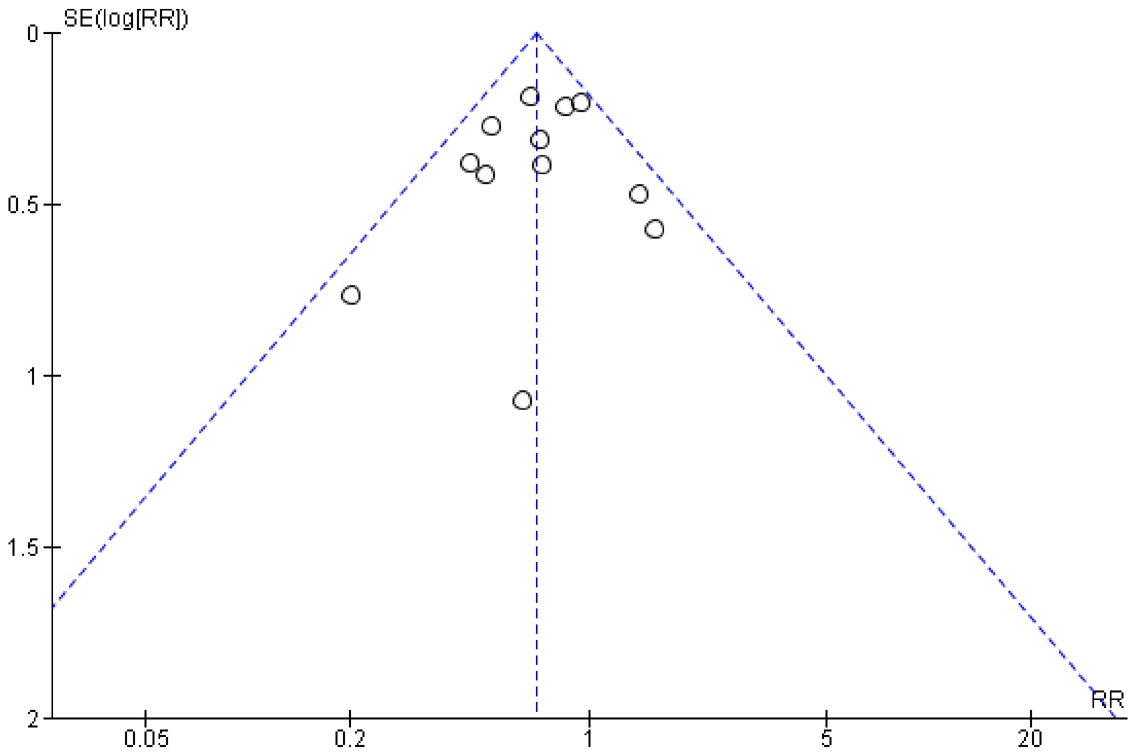

Figure S6. Funnel plot assessing publication bias in last-follow up-mortality. Each circle represents an individual study. The vertical dashed line indicates the pooled risk ratio from the meta-analysis, and the diagonal lines represent pseudo 95% confidence limits. Visual inspection suggests slight asymmetry; however, Egger's regression test did not demonstrate statistically significant small-study effects (p = 0.56).

Study or Subgroup	MIS Mean	MIS SD	MIS Total	MM Mean	MM SD	MM Total	Weight	Mean Difference IV, Random, 95% CI	Year
Teernstra 2003	3.9	6.1	36	1.8	7.1	34	9.7%	2.10 [-1.01, 5.21]	2003
Hanley 2016	9.25	1.98	54	8.5	1.839	42	25.5%	0.75 [-0.02, 1.52]	2016
Hanley 2019	11	1.78	250	10.15	1.96	249	27.8%	0.85 [0.52, 1.18]	2019
Pradilla 2024	6.9	6.8	150	9.7	7.6	150	18.6%	-2.80 [-4.43, -1.17]	2024
Arthur 2025	5.9	5	154	7.3	6.7	82	18.4%	-1.40 [-3.05, 0.25]	2025
**Total (95% CI)**			**644**			**557**	**100.0%**	**-0.15 [-1.34, 1.05]**	

Heterogeneity: Tau² = 1.30; Chi² = 25.41, df = 4 (P < 0.0001); I² = 84%
Test for overall effect: Z = 0.24 (P = 0.81)

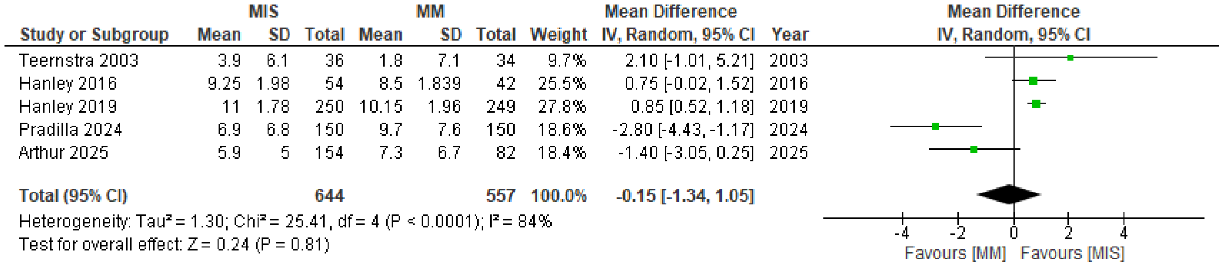

Figure S7. Forest plot of ICU length of stay comparing minimally invasive surgery (MIS) with medical management (MM). High heterogeneity was observed (I^2 = 84%), which was not resolved by leave-one-out analysis.

Study or Subgroup	MIS Mean	MIS SD	MIS Total	MM Mean	MM SD	MM Total	Weight	Mean Difference IV, Random, 95% CI	Year
**8.1.2 stereotactic aspiration ± thrombolytics**									
Teernstra 2003	3.9	6.1	36	1.8	7.1	34	9.7%	2.10 [-1.01, 5.21]	2003
Hanley 2016	9.25	1.98	54	8.5	1.839	42	25.5%	0.75 [-0.02, 1.52]	2016
Hanley 2019	11	1.78	250	10.15	1.96	249	27.8%	0.85 [0.52, 1.18]	2019
**Subtotal (95% CI)**			**340**			**325**	**63.0%**	**0.85 [0.55, 1.15]**	
Heterogeneity: Tau² = 0.00; Chi² = 0.69, df = 2 (P = 0.71); I² = 0%									
Test for overall effect: Z = 5.52 (P < 0.00001)									
**8.1.3 Endoscopic evacuation**									
Pradilla 2024	6.9	6.8	150	9.7	7.6	150	18.6%	-2.80 [-4.43, -1.17]	2024
Arthur 2025	5.9	5	154	7.3	6.7	82	18.4%	-1.40 [-3.05, 0.25]	2025
**Subtotal (95% CI)**			**304**			**232**	**37.0%**	**-2.11 [-3.48, -0.73]**	
Heterogeneity: Tau² = 0.28; Chi² = 1.40, df = 1 (P = 0.24); I² = 28%									
Test for overall effect: Z = 3.01 (P = 0.003)									
**Total (95% CI)**			**644**			**557**	**100.0%**	**-0.15 [-1.34, 1.05]**	

Heterogeneity: Tau² = 1.30; Chi² = 25.41, df = 4 (P < 0.0001); I² = 84%
Test for overall effect: Z = 0.24 (P = 0.81)
Test for subgroup differences: Chi² = 16.97, df = 1 (P < 0.0001), I² = 94.1%

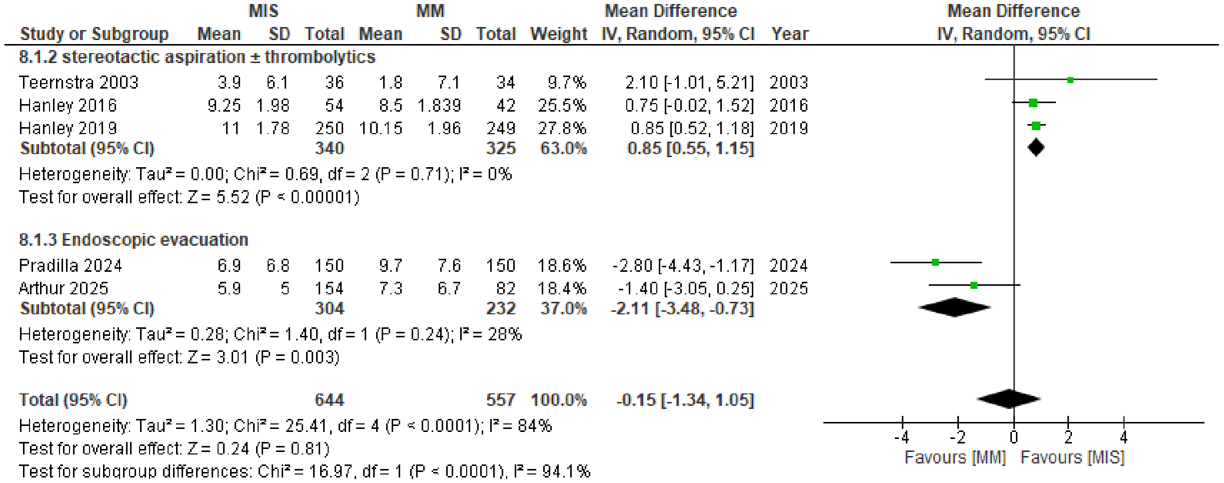

Figure S8. Forest plot of ICU length of stay subgrouped by MIS technique. Stereotactic aspiration with local thrombolytics was associated with longer ICU stay, whereas endoscopic evacuation was associated with shorter ICU stay.

	MIS		MM			Risk Ratio	
Study or Subgroup	Events	Total	Events	Total	Weight	M-H, Random, 95% CI	Year
Auer 1989	2	50	15	50	10.7%	0.13 [0.03, 0.55]	1989
Teernstra 2003	7	36	0	34	4.1%	14.19 [0.84, 239.28]	2003
Kim 2009	14	204	9	183	16.8%	1.40 [0.62, 3.15]	2009
Wang 2009	19	195	9	182	17.3%	1.97 [0.92, 4.24]	2009
Hanley 2016	5	54	1	42	6.5%	3.89 [0.47, 32.04]	2016
Vespa 2016	3	14	5	42	11.8%	1.80 [0.49, 6.59]	2016
Hanley 2019	6	255	3	251	11.1%	1.97 [0.50, 7.79]	2019
Deng 2022	0	39	1	39	3.4%	0.33 [0.01, 7.94]	2022
Guo 2024	4	61	3	61	10.5%	1.33 [0.31, 5.71]	2024
Pradilla 2024	3	150	0	150	3.8%	7.00 [0.36, 134.36]	2024
Sun 2025	4	170	0	170	3.9%	9.00 [0.49, 165.88]	2025
**Total (95% CI)**		**1228**		**1204**	**100.0%**	**1.57 [0.84, 2.97]**	
Total events	67		46				

Heterogeneity: Tau² = 0.45; Chi² = 18.49, df = 10 (P = 0.05); I² = 46%
Test for overall effect: Z = 1.40 (P = 0.16)

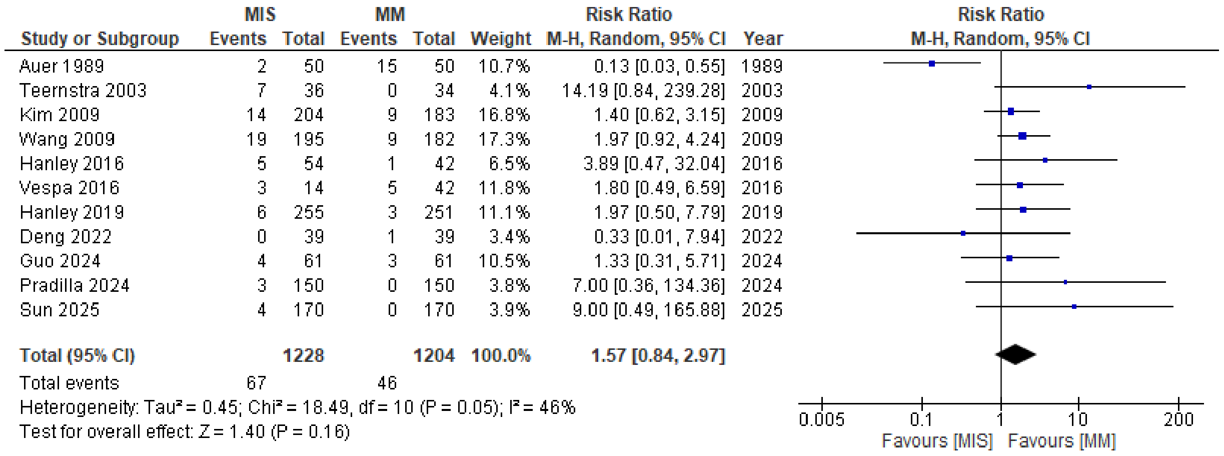

Figure S9. Forest plot of rebleeding risk comparing minimally invasive surgery (MIS) with medical management (MM). The pooled analysis showed a numerically higher risk in the MIS group; however, the difference was not statistically significant.

Study or Subgroup	MIS Events	MIS Total	MM Events	MM Total	Weight	Risk Ratio M-H, Random, 95% CI	Year
Teernstra 2003	20	36	24	34	9.8%	0.79 [0.55, 1.13]	2003
Vespa 2016	3	14	6	42	0.8%	1.50 [0.43, 5.22]	2016
Hanley 2016	5	54	1	42	0.3%	3.89 [0.47, 32.04]	2016
Deng 2022	2	39	5	39	0.5%	0.40 [0.08, 1.94]	2022
Pradilla 2024	95	150	118	150	59.5%	0.81 [0.69, 0.93]	2024
Arthur 2025	80	152	56	82	29.1%	0.77 [0.62, 0.95]	2025
**Total (95% CI)**		**445**		**389**	**100.0%**	**0.80 [0.71, 0.89]**	
Total events	205		210				

Heterogeneity: $Tau^2 = 0.00$; $Chi^2 = 4.05$, df = 5 (P = 0.54); $I^2 = 0\%$

Test for overall effect: Z = 3.89 (P = 0.0001)

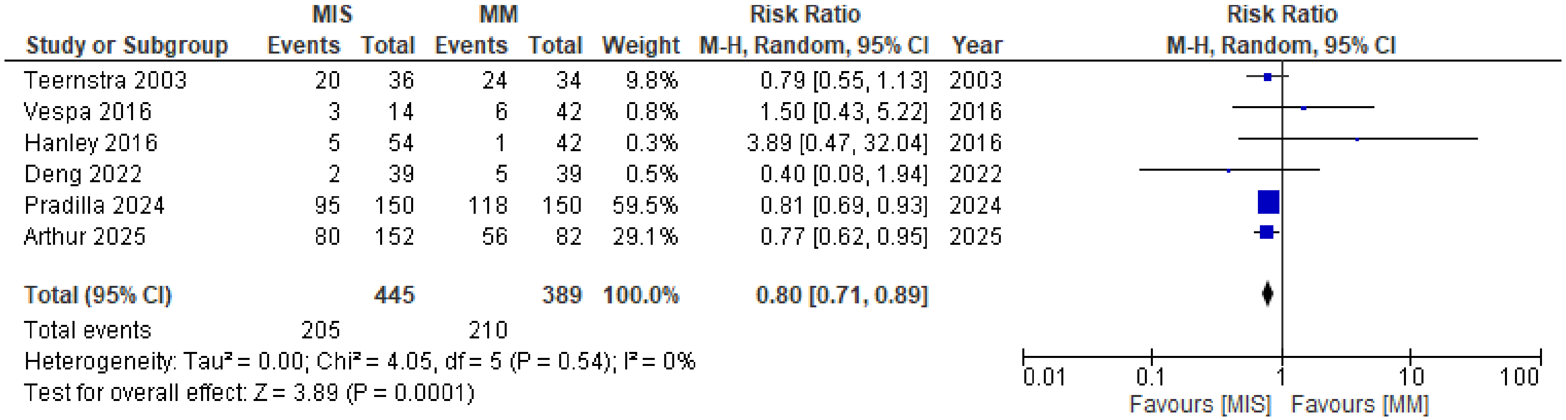

Figure S10. Forest plot of severe adverse events comparing minimally invasive surgery (MIS) with medical management (MM). The overall analysis favored MIS.
